# Epithelial–mesenchymal transition in pulmonary fibrosis: molecular mechanisms and emerging therapeutic strategies

**DOI:** 10.3389/fmed.2025.1658001

**Published:** 2025-09-08

**Authors:** Weiyi Li, Yinghai Xie, Zhenzhen Chen, Dongli Cao, Yu Wang

**Affiliations:** ^1^School of Medicine, Anhui University of Science and Technology, Huainan, China; ^2^Key Laboratory of Industrial Dust Deep Reduction and Occupational Health and Safety of Anhui Higher Education Institutes, Huainan, China; ^3^The First Affiliated Hospital of Anhui University of Science and Technology (Huainan First People's Hospital), Huainan, China; ^4^Anhui Province Engineering Laboratory of Occupational Health and Safety, Huainan, China

**Keywords:** epithelial–mesenchymal transition, pulmonary fibrosis, ferroptosis, metabolic reprogramming, mechanical stress, antifibrotic therapy

## Abstract

Pulmonary fibrosis is a progressive lung-scarring disease for which curative options remain limited. This review examines how epithelial–mesenchymal transition (EMT) contributes to fibrotic remodeling in subsets of pulmonary fibrosis (PF), delineates where the evidence is strongest, and highlights emerging therapeutic directions. PF encompasses idiopathic PF (IPF) and diverse non-IPF interstitial lung diseases driven by autoimmunity, exposures, or genetics, in which EMT involvement is variable. Recent laboratory and clinical work has been analyzed and the evidence grouped into four areas: well-known growth-factor signals; immune and inflammatory crosstalk; newer drivers such as iron-linked cell death, metabolic change and tissue stretch; and emerging medicines that temper these pathways, including licensed antifibrotics, experimental small molecules, natural compounds and RNA-based agents. Collectively, EMT emerges as a potentially reversible hub linking epithelial stress to scar formation, suggesting stage-specific combination strategies supported by single-cell profiling, lung organoids, and targeted delivery.

## 1 Introduction

Epithelial-mesenchymal transition (EMT) is defined as a complex cellular program in which epithelial cells lose polarity and intercellular adhesion while acquiring the morphology and functional traits of mesenchymal cells, manifested by enhanced migration, invasion, and resistance to apoptosis (1). Key features encompass reduced expression of adhesion molecules such as E-cadherin, cytoskeletal reconstruction indicated by elevated vimentin and N-cadherin, basement-membrane breakdown mediated by activated matrix metalloproteinases, and consequent enhancement of cell motility (2).

During the course of pulmonary fibrosis (PF), EMT is thought to exert a central regulatory role by reshaping alveolar epithelial cell phenotypes and activating fibroblasts (3, 4). Within this pathological context, alveolar epithelial cells—particularly type II alveolar epithelial cells (ATII)—are observed to transdifferentiate into myofibroblast-like cells after EMT, becoming a principal source of excessive extracellular matrix (ECM) deposition; this process is accompanied by down-regulation of the epithelial marker E-cadherin and up-regulation of mesenchymal markers vimentin and N-cadherin, thereby aggravating EMT and accelerating fibrosis progression (5). Consequently, EMT is widely implicated in PF pathogenesis as a bridge between epithelial injury and fibrotic remodeling. However, its pathogenic weight likely varies across PF subtypes and disease stages (6).

Pulmonary fibrosis encompasses a spectrum of progressive chronic interstitial lung disorders, of which idiopathic pulmonary fibrosis (IPF) is the archetypal form, distinguished by repeated alveolar-epithelial insults, dysregulated EMT, and excessive fibroblast activation, leading to architectural derangement and relentless functional decline. Epidemiological evidence indicates that this disease has a poor prognosis and short survival. However, 30–50% of patients with non-IPF patients eventually develop progressive pulmonary fibrosis (PPF), the clinical burden of which parallels that of IPF (7). Although pirfenidone and nintedanib delay functional deterioration in certain cases, they fail to halt or reverse fibrogenesis and are further limited by variable efficacy, drug resistance, and liver-kidney toxicities. Beyond IPF, EMT also plays a crucial role in non-IPF fibrotic ILDs, including silicosis (silica exposure) (8), rheumatoid-arthritis-associated ILD (RA-ILD) (9), and radiation-induced pulmonary fibrosis (10). However, IPF exhibits marked differences in etiology and immunopathological mechanisms compared with non-IPF fibrotic ILDs driven by immune mediation or antigen exposure (11, 12). Pulmonary fibrosis is not restricted to chronic inhalational injury; in addition to IPF, it encompasses immune-mediated, exposure-related, and genetic forms with differing initiating mechanisms and degrees of EMT involvement. In this review, PF is used as a general term that includes both IPF and non-IPF fibrotic ILDs. When mechanisms specific to IPF are discussed, they are stated explicitly. When not specified, the discussion refers to mechanisms that are common to PF as a whole.

Although EMT is a well-documented process in various cancers, its occurrence in pulmonary fibrosis (PF) follows a distinct trajectory driven by chronic epithelial injury, aberrant repair, and matrix remodeling. Current studies in PF face key gaps, including the lack of standardized, dynamic EMT assessment, limited translational relevance of acute injury models to human idiopathic PF, and insufficient integration of emerging mechanisms such as ferroptosis, metabolic reprogramming, and mechano-signaling. This review addresses these gaps by providing a PF-specific EMT framework that integrates classical and novel pathways, re-maps therapeutic strategies to EMT nodes, and highlights methodological advances that may facilitate clinical translation. Elucidating the regulatory mechanisms of EMT in pulmonary fibrosis has substantial clinical implications: on one hand, EMT constitutes the common pathway linking various fibrogenic stimuli (e.g., inflammation, oxidative stress, mechanical tension) to end-stage fibrosis; on the other, targeting pivotal EMT nodes may offer breakthrough strategies to reverse fibrosis. This review systematically summarizes the role of EMT in pulmonary fibrogenesis from four perspectives—molecular mechanisms, microenvironmental regulation, emerging pathways, and therapeutic strategies—and discusses the challenges and future directions in this field.

## 2 Key regulatory mechanisms of EMT

### 2.1 Central role of TGF-β superfamily signaling

As a pleiotropic cytokine, transforming growth factor-β (TGF-β) exerts pivotal regulatory functions in pulmonary fibrosis via both Smad-dependent and Smad-independent pathways, driving epithelial–mesenchymal transition (EMT) and promoting airway inflammation and remodeling (13). In the Smad-dependent pathway, activated TGF-β phosphorylates Smad2/3, which form complexes with Smad4 and translocate to the nucleus to induce EMT-related transcription factors (Snail, Slug, Twist, ZEB1/2) (14). These factors repress epithelial markers (E-cadherin, ZO-1) while upregulating mesenchymal markers (α-SMA, N-cadherin, vimentin), leading to myofibroblast differentiation and excessive extracellular matrix (ECM) deposition (15). Li et al. demonstrated that BFHX attenuates bleomycin-induced EMT in mice by inhibiting Smad2/3 phosphorylation and blocking fibroblast-to-myofibroblast transition (16).

The TGF-β/Smad cascade also contributes to airway fibrosis through induction of amphiregulin (AREG), which activates EGFR signaling to enhance fibroblast proliferation and myofibroblast conversion. In TGF-β-driven NSCLC models, loss of Smad3 disrupts SMAD3/SMAD4 complex formation, suppresses Snail expression, and attenuates ECM accumulation, thereby reducing EMT and fibrosis (17, 18). Similarly, Smad3 silencing decreases AREG expression and blocks the downstream EGFR/JNK/AP-1 pathway, lowering EMT markers such as CCN2 and fibronectin (19).

Beyond Smad signaling, EMT is regulated by non-Smad pathways including MAPK (ERK, JNK, p38), PI3K/AKT, and Rho-GTPases (20, 21). The PI3K/AKT pathway is particularly critical and can be activated by multiple factors, notably galectin-3 (LGALS3) and heparanase. In a silica-induced pulmonary fibrosis, galectin-3 (LGALS3) markedly promotes endothelial-to-mesenchymal transition (EndMT) through activation of the PI3K/AKT signaling pathway. This is evidenced by the down-regulation of VE-cadherin and the up-regulation of N-cadherin, α-SMA, and Snail, ultimately exacerbating fibrotic progression (22). In idiopathic pulmonary fibrosis, heparanase is elevated in M2-polarized macrophages, where it drives PI3K/AKT-dependent autophagy and indirectly facilitates EMT. Inhibiting heparanase with OGT2115 improves survival and reduces fibrosis in bleomycin-treated mice (23).

### 2.2 Inflammatory and tumor microenvironment (TME)

Recent reviews and experimental studies have demonstrated that signaling from the tumor microenvironment actively drives EMT, thereby promoting tumor cell invasion and metastasis (24, 25). In the course of pulmonary fibrosis, sustained activation of innate and adaptive immunity leads to infiltration of diverse immune cells into lung tissue, establishing a complex immune microenvironment. Macrophages, lymphocytes, neutrophils and others secrete cytokines, chemokines and bioactive mediators that spatiotemporally coordinate the initiation and maintenance of EMT. Dynamic shifts in immune-cell states, particularly macrophage polarization and inflammasome activation markedly influence the stability and plasticity of alveolar-epithelial phenotypes, ultimately fostering mesenchymal conversion. Given the centrality of the inflammatory and immune microenvironment in EMT regulation, recent advances and mechanistic insights in pulmonary fibrosis are summarized in the following section.

Within the TME, tumor-associated macrophages (TAMs) are highly plastic immune cells that can differentiate into either classical M1 or alternative M2 phenotypes. As previously discussed, alternatively activated M2 macrophages exert fibrogenic effects in the lung and show strong connections with EMT. Recent work indicates that ATII cells play a pivotal role in the comorbid pathogenesis of IPF and lung cancer-associated fibrosis (26). Additional studies show that M2-polarized TAMs secrete miR-155-5p-rich exosomes (M2-exos) that, in a HuR-dependent manner, activate the VEGFR2/PI3K/AKT/mTOR cascade, enhancing proliferation, migration and EMT, and ultimately promoting NSCLC malignancy (27).

The NLRP3 inflammasome, a critical intracellular multiprotein complex of innate immunity, can be activated by danger signals such as silica (SiO_2_). Evidence shows that silica exposure markedly up-regulates NLRP3, Caspase-1 and IL-1β in human bronchial epithelial (HBE) cells and elevates the release of pro-inflammatory IL-1β and IL-18. Such inflammatory mediators activate fibroblasts, enhance their proliferation, and foster collagen and ECM accumulation, thereby instigating chronic inflammation and serving as major engines of fibrogenesis. Continued inflammatory insult additionally stimulates EMT-regulatory transcription factors, eliciting EMT marked by increased α-SMA and reduced E-cadherin expression (28). Notably, the NLRP3 inhibitor MCC950 or the Caspase-1 blocker Z-YVAD-FMK markedly reduces NLRP3, Caspase-1 and α-SMA levels while restoring E-cadherin, thereby suppressing EMT and alleviating lung inflammation and fibrosis (29).

Chemotactic factors bind specific receptors to direct immune-cell migration, governing inflammatory recruitment, niche formation and tissue restructuring. In pulmonary fibrosis, chemokines not only sustain inflammatory-cell recruitment but also induce EMT and activate fibroblasts, directly advancing fibrotic progression. Studies show that the chemokine CCL1, derived from alveolar macrophages, is aberrantly up-regulated in pulmonary fibrosis; binding of CCL1 to autocrine motility factor receptor (AMFR) on lung fibroblasts activates the AMPKα-FOXO3–Snail axis, driving fibroblast-to-myofibroblast differentiation and Snail-mediated EMT, thereby accelerating fibrosis (30). Genetic ablation of macrophage CCL1 or AMFR, or therapeutic neutralization of CCL1, substantially relieves fibrotic pathology. Follow-up experiments corroborate these findings, demonstrating that targeting the CCL1–AMFR axis significantly ameliorates pulmonary fibrosis. Importantly, heightened activation of the CCL1–AMFR pathway in lung tissue drives both inflammatory responses, EMT and ECM deposition, uncovering a promising therapeutic avenue against pulmonary fibrosis (31).

### 2.3 Other pivotal signaling pathways

The Wnt/β-catenin pathway exerts a decisive regulatory role in pulmonary fibrosis (32). By inhibiting GSK-3β activity, this pathway stabilizes β-catenin, facilitates its nuclear translocation and enables formation of a β-catenin/TCF–LEF transcriptional complex, which cooperates with TGF-β to trigger EMT in alveolar epithelial cells (33). Wnt binding to Frizzled receptors suppresses GSK-3β, prevents β-catenin degradation, elevates intracellular β-catenin and up-regulates Snail, thereby advancing EMT in pulmonary fibrosis and cancer-related disorders (34, 35). Early studies demonstrated that targeting Wnt/β-catenin signaling suppresses Snail1-driven mesenchymal markers (vimentin, N-cadherin) and ameliorates fibrosis in silicosis models (36). Notably, bone-marrow-derived mesenchymal stem cells (MSCs) and their microvesicles (MSC-MVs) modulate Wnt/β-catenin signaling, markedly down-regulate the mesenchymal marker α-SMA and confer protection against early ARDS-related lung fibrosis (37, 38).

Notch signaling, activated through ligand–receptor engagement, participates in EMT regulation and forms a feedback loop with TGF-β/Smad pathways (39, 40). Recent investigations have delineated the multifaceted involvement of Notch signaling in pulmonary fibrosis. Kana et al. revealed a bidirectional circuit between TGF-β-Smad and Notch signaling: TGF-β activates Notch to induce α-SMA, while Notch activation further represses E-cadherin and elevates mesenchymal markers (41). Moreover, SiO_2_ challenge elevates the p53/RMRP/miR-122 pathway, thereby engaging Notch signaling, promoting EMT and intensifying fibrotic responses (42). Mechanistic studies indicate that Notch1 over-expression augments Smad phosphorylation, drives myofibroblast differentiation and promotes pro-inflammatory cytokine release (43). Therapeutically, natural compounds such as emodin inhibit neutrophil elastase, block Notch1 activation, alleviate TGF-β-induced EMT and exhibit anti-fibrotic activity (44).

Dysregulated activation of the Hippo–YAP/TAZ cascade represents a major cause of defective alveolar epithelial regeneration and intensified EMT, with YAP (Yes-associated protein) and TAZ (transcriptional co-activator with PDZ-binding motif) acting as its pivotal downstream effectors (45). In alveolar epithelial cells from patients with pulmonary fibrosis, YAP/TAZ display persistent nuclear localization, this aberrant activation correlates closely with impeded differentiation of type-II alveolar epithelial cells (AT2) into type-I cells (AT1), culminating in failed alveolar regeneration. Concomitantly, activated YAP/TAZ cooperate with TGF-β signaling to potentiate Smad-dependent transcription, up-regulating EMT-driving transcription factors such as Snail and Twist, resulting in loss of epithelial polarity, increased α-SMA and N-cadherin expression, further fibroblast activation and extracellular-matrix (ECM) accumulation, thereby accelerating fibrosis. Importantly, increased matrix stiffness and augmented tensile stress within fibrotic lung tissue serve as key mechanical cues that sustain YAP/TAZ activation, these exogenous physical signals drive their nuclear translocation, maintain abnormal transcriptional activity, disrupt the normal regeneration–repair equilibrium, and thus foster pathological fibrotic remodeling (46).

**Figure 1:**
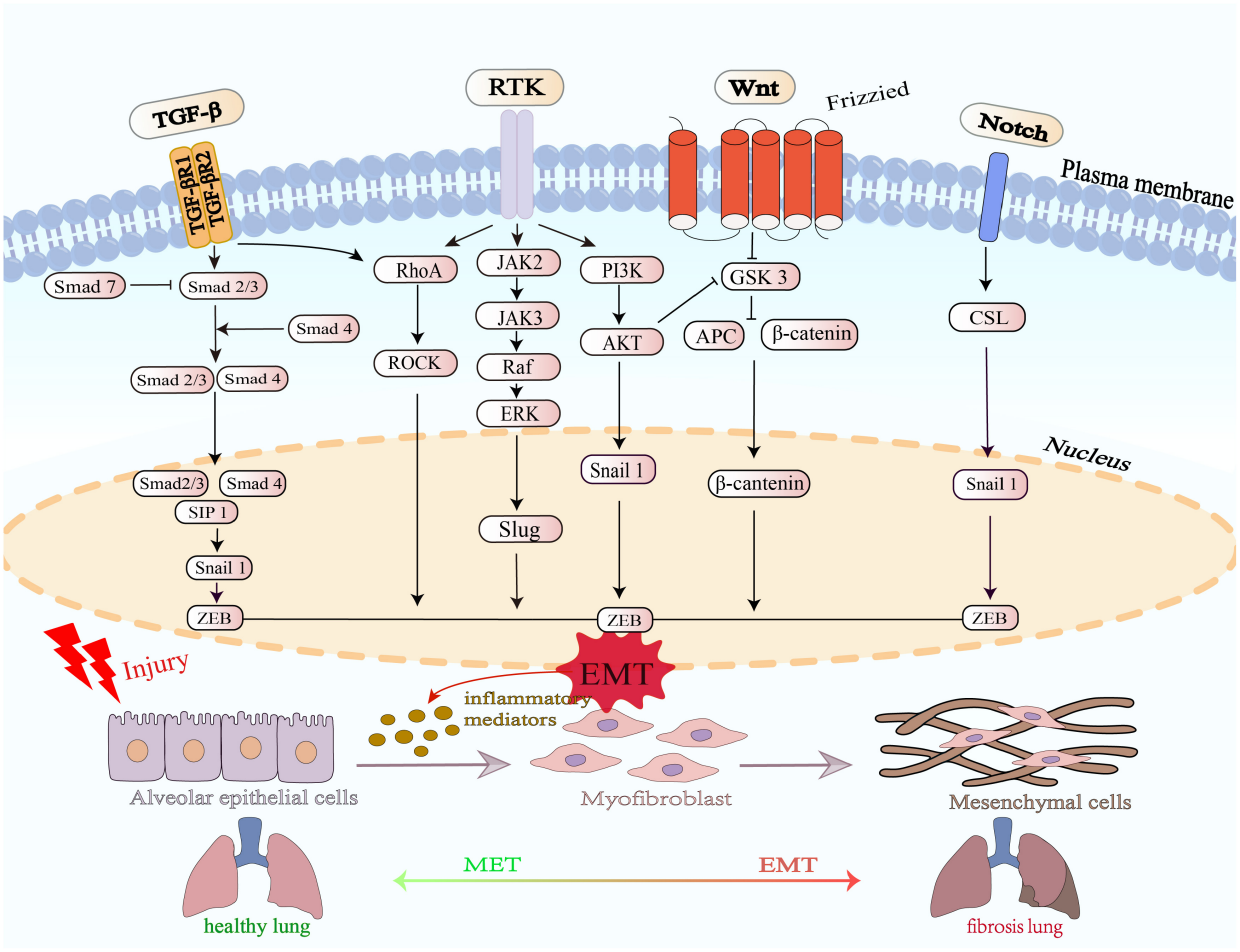
Schematic illustration of the molecular mechanisms by which epithelial–mesenchymal transition (EMT) regulates the pathogenesis of pulmonary fibrosis. The TGF-β, Wnt, PI3K/AKT, MAPK, Rho-GTPase and Notch signaling pathways cooperatively promote EMT by activating downstream transcription factors such as Snail, SIP1, and ZEB. Upon injury, alveolar epithelial cells undergo EMT under the influence of inflammatory mediators, leading to the generation of myofibroblasts and mesenchymal cells, which contribute to extracellular matrix deposition and fibrotic remodeling. The balance between mesenchymal–epithelial transition (MET) and EMT determines the progression from a healthy lung to fibrotic lung tissue.

The receptor for advanced glycation end-products (RAGE) is a multi-ligand receptor predominantly expressed on type-II alveolar epithelial (AT2) cells, and its ligand HMGB-1 is highly up-regulated during pulmonary fibrosis. Accumulating evidence indicates that RAGE is essential for preserving the structural integrity, barrier function, and differentiation status of the normal alveolar epithelium (47). Conversely, in the fibrotic lung, RAGE expression is markedly reduced; its deficiency correlates with alveolar destruction, enhanced inflammatory responses and fibroblast activation, and RAGE-knockout models reveal that loss of the receptor exacerbates epithelial-mesenchymal transition (EMT) of alveolar epithelial cells, thereby promoting parenchymal remodeling and collagen deposition (48). In contrast, pharmacological or genetic activation of RAGE signaling suppresses TGF-β1-induced expression of Snail and Twist, consequently slowing fibrogenic progression. Moreover, the RAGE–HMGB1 complex exerts regulatory functions during lung injury and repair, yet chronic activation of this axis may amplify inflammation and fibrosis (49). Animal studies confirm that blockade of RAGE or HMGB1 signaling significantly reduces collagen deposition and inflammation in lung tissue, highlighting this pathway as a potential therapeutic target for pulmonary fibrosis. A schematic summary of the major EMT-regulatory pathways—including TGF-β/Smad, PI3K/AKT, Wnt/β-catenin, Notch, Hippo–YAP/TAZ, and associated inflammatory mediators—is shown in Figure 1.

### 2.4 Epigenetic regulation

Non-coding RNAs (ncRNAs) are functionally broadly classified structural RNAs (e.g., rRNA, tRNA) and regulatory ncRNAs, the latter including microRNAs (miRNAs), circular RNAs (circRNAs) and small interfering RNAs (siRNAs). Accumulating evidence indicates that regulatory ncRNAs exert critical effects on pulmonary fibrosis through epigenetic regulation, post-transcriptional modulation and signaling pathways, and are intimately intertwined with EMT dynamics (50, 51).

In fibrotic disorders, miRNAs influence disease progression through multiple mechanisms that drive fibroblast–myofibroblast differentiation, disrupt extracellular matrix (ECM) homeostasis, and coordinate the inflammatory–fibrotic cascade. Among EMT-related miRNAs, miR-34c-5p directly targets *FOSL1* (Fra-1) to reverse silica-induced EMT, restoring E-cadherin expression while suppressing vimentin and reducing cell proliferation and migration (52). miR-21 remains a prototypical EMT-promoting miRNA; it is markedly upregulated in idiopathic pulmonary fibrosis (IPF) lungs and fibrotic mouse lungs, predominantly localized to myofibroblasts, and inhibition via antimiR-21 therapy attenuates fibrosis (53, 54).

In the context of circRNAs, several molecules have been identified as upstream modulators of EMT and fibroblast activation. For instance, in silica-induced pulmonary fibrosis, circRNA CDR1as acts as a sponge for miR-7, relieving TGFBR2 from repression and thereby promoting EMT and accelerating fibrosis (55). Similarly, circMKLN1 suppresses miR-26a/b, resulting in increased CDK8 expression, enhanced EMT, and exacerbated fibrotic progression (56). These mechanistic insights underscore both the regulatory importance of circRNAs in EMT and their potential as therapeutic targets.

Targeted gene-silencing approaches have also been shown to directly modulate EMT. For example, siRNA against osteopontin (OPN) significantly inhibited EMT in bleomycin (BLM)-induced murine pulmonary fibrosis, as evidenced by increased E-cadherin, decreased vimentin, and reduced fibrotic remodeling (57). Likewise, HDAC3 siRNA suppressed hypoxia-induced EMT in alveolar epithelial cells via the miR-224/FOXA1 axis, leading to reduced fibrosis severity (58).

Collectively, these representative studies illustrate the diverse mechanisms by which ncRNAs influence EMT in pulmonary fibrosis. However, they constitute only a fraction of the molecular network underlying this process. Ongoing high-throughput transcriptomic profiling, coupled with advances in RNA delivery technologies, is expected to uncover additional ncRNA targets with potential therapeutic relevance, further expanding the scope for precision interventions in EMT-driven fibrotic lung disease.

## 3 Emerging mechanisms regulating EMT

### 3.1 Ferroptosis and oxidative stress

In pulmonary fibrosis (PF), ferroptosis exhibits a significant interplay with EMT, with the most compelling evidence observed in IPF. This interaction is driven by recurrent epithelial injury, oxidative stress, and inflammatory amplification (59, 60). Evidence indicates that repeated injury to alveolar epithelial cells is recognized as a pivotal early pathogenic event in IPF, triggered by exogenous insults such as environmental toxins, pathogen infection or specific medications. Under these conditions, intracellular ferrous iron (Fe^2^^+^) overload, elevated reactive oxygen species (ROS) and weakened antioxidant defenses markedly enhance the susceptibility of type-II alveolar epithelial cells (ATII) to ferroptosis. During early disease, stimuli such as bleomycin (BLM) or lipopolysaccharide (LPS) induce ferroptosis in ATII cells, disrupting the alveolar barrier and intensifying oxidative stress (61). Concomitant liberation of inflammatory and fibrogenic factors, particularly TGF-β activation, provides a major impetus for EMT onset. During fibrotic progression, TGF-β activates a TAZ–TEAD program that elevates TFRC, increases intracellular Fe^2^^+^ in fibroblasts and promotes their myofibroblastic conversion, maintaining EMT-linked features (62). Notably, despite heightened iron burden, fibroblasts evade ferroptosis by invoking iron-storage pathways and lipid-metabolic reprogramming, thereby preserving active mesenchymal functions (63).

Ferroptosis aggravates epithelial injury and, by generating ROS and activating pro-inflammatory pathways such as TGF-β and NF-κB, further promotes EMT, mesenchymal activation and tissue remodeling (61). Given its central role in IPF, ferroptosis has emerged as a promising therapeutic target. Experimental data show that ferrostatin-1 and liproxstatin-1, which block lipid peroxidation, significantly reduce epithelial injury, collagen build-up and ROS accumulation in BLM models (64, 65). The iron chelator deferoxamine (DFO) removes labile iron, curtails Fenton chemistry and thereby blocks ferroptotic signaling. The antioxidant N-acetyl-L-cysteine (NAC) boosts glutathione (GSH), preserves GPX4 activity and indirectly restrains both ferroptosis and EMT (66). Collectively, these findings indicate that ferroptosis and EMT share oxidative stress as a common pathological denominator and can be concurrently mitigated via overlapping therapeutic targets, offering novel strategies for IPF management.

**Figure 2:**
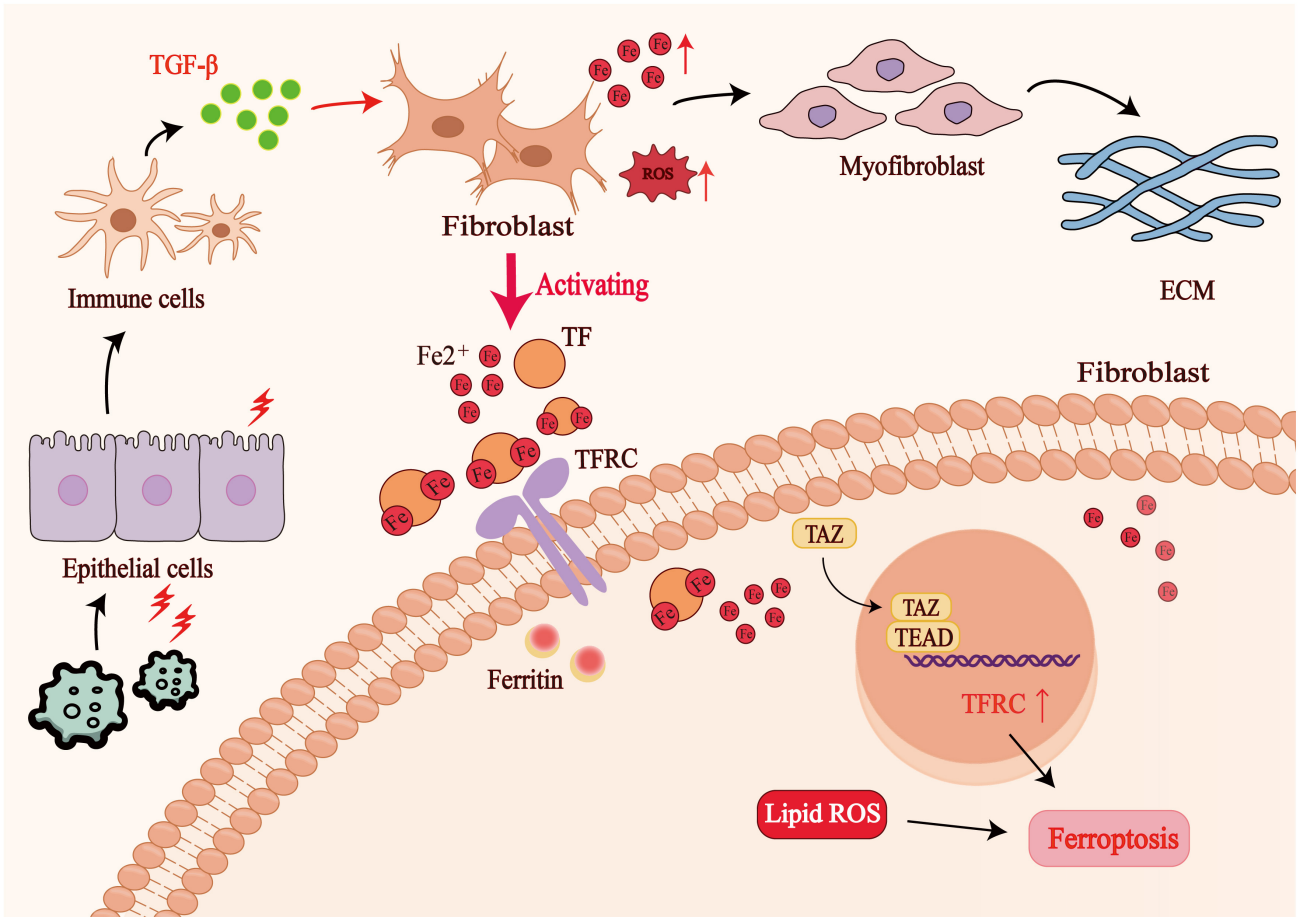
Crosstalk between ferroptosis and EMT in pulmonary fibrosis. Recurrent epithelial injury and Fe^2^^+^ overload trigger excessive ROS production and ferroptosis in alveolar epithelial cells, accompanied by activation of TGF-β signaling and release of profibrotic mediators. Oxidative stress and inflammatory signaling up-regulate EMT transcription factors (Snail, Slug, Twist), driving epithelial dedifferentiation. In fibroblasts, the TAZ–TEAD program elevates TFRC expression, increases iron uptake and promotes myofibroblast conversion, while ferritin and lipid metabolic reprogramming protect fibroblasts from ferroptosis. These interactions establish a self-reinforcing loop of iron dysregulation, EMT, and oxidative stress that perpetuates fibrotic remodeling.

It is noteworthy that aberrant iron homeostasis forms the central hub of ferroptosis-EMT crosstalk. Excess Fe^2^^+^ drives hydroxyl-radical generation via the Fenton reaction, escalates ROS and activates TGF-β and NF-κB pathways, elevating Snail, Slug and Twist, and inducing loss of polarity and mesenchymal acquisition in epithelial cells. Concurrently, EMT involves metabolic reprogramming with increased iron uptake (TFR1 up-regulation), further accumulating Fe^2^^+^ and amplifying oxidative stress. Thus, iron-metabolic dysregulation promotes EMT, which in turn exacerbates iron imbalance, establishing a self-reinforcing positive-feedback loop that drives relentless IPF progression and fibrotic remodeling (67). A schematic overview of the ferroptosis–EMT crosstalk and its contribution to pulmonary fibrosis is presented in Figure 2, which highlights the key molecular drivers and the self-reinforcing feedback loop between iron dysregulation, oxidative stress, and EMT.

### 3.2 Metabolic reprogramming

During EMT of alveolar epithelial cells, the metabolic profile shifts from oxidative phosphorylation (OXPHOS) to aerobic glycolysis, closely mirroring the classical Warburg effect observed in cancer cells (68). EMT inducers (TGF-β, Snail and Twist) markedly increase glycolytic flux by up-regulating pivotal enzymes such as hexokinase-2 (HK2), 6-phosphofructo-2-kinase (PFKFB3) and lactate dehydrogenase-A (LDHA). Excess lactate generated by heightened glycolysis acidifies the extracellular milieu and activates TGF-β signaling, thereby amplifying EMT (69). Monocarboxylate transporter-1 (MCT1) is the principal carrier governing lactate trans-membrane movement and thus preserves extracellular lactate homeostasis (70). Lipopolysaccharide (LPS) suppresses MCT1 expression, hindering lactate efflux and resulting in its excessive accumulation outside the cell. In the absence of MCT1, even moderate lactate levels can trigger EMT, implying that lactate-handling defects are pivotal EMT drivers. Subsequent work demonstrates that MCT1 over-expression restores lactate homeostasis, reverses LPS-induced EMT, mitigates alveolar destruction and collagen deposition, and markedly attenuates pulmonary fibrosis. Overall, glycolysis and EMT create a positive-feedback loop that both accelerates epithelial mesenchymalization and augments fibroblast activity together with matrix deposition. Heightened glycolysis and its by-product lactate serve not only as metabolic hallmarks of pulmonary fibrosis but also as central engines propelling EMT and disease advancement.

During EMT, metabolic re-programming extends beyond glycolytic enhancement to a pronounced reliance on glutamine metabolism for energy generation and biosynthetic precursors. Glutamine, the most abundant non-essential amino acid, contributes to protein and nucleotide synthesis, energy metabolism and redox balance, and plays vital regulatory roles in cancer, inflammation and tissue remodeling. Multiple studies indicate that glutamine catabolism activates TGF-β signaling, induces Snail and Twist, and supplies metabolites such as α-ketoglutarate (α-KG) that facilitate epigenetic regulation of EMT genes (71). In bleomycin-induced murine fibrosis, glutamine metabolism drives up-regulation of fibrotic genes such as COL1A1 and α-SMA. TGF-β stimulation up-regulates glutaminase-1 (GLS1) in lung fibroblasts and epithelial cells, intensifying glutamine conversion and stimulating cell migration, proliferation and collagen deposition. Moreover, α-KG, a glutamine-derived intermediate, modulates histone demethylases, epigenetically potentiating EMT and synergizing with fibrotic progression (72). Targeting glutamine metabolism holds therapeutic promise: the GLS1 inhibitor CB-839 efficiently blocks TGF-β-driven collagen synthesis *in vitro* and significantly alleviates fibrosis severity and lung function decline in BLM models (73).

Throughout pulmonary fibrogenesis, re-programming of fatty-acid metabolism has emerged as a key metabolic determinant of alveolar epithelial homeostasis and EMT regulation (74). Recent evidence suggests that defective fatty-acid metabolism within type-II alveolar cells may act as an early trigger of fibrosis (75). Using AT2-specific knockout mice lacking the lipid-metabolic enzymes Scd1 and Elovl6, investigators observed spontaneous pulmonary fibrosis. Mechanistically, Scd1/Elovl6 deficiency provokes a canonical EMT phenotype in AT2 cells, characterized by E-cadherin loss and increased Vimentin and Zeb1. The deletions also disrupt mitochondrial architecture, accumulate ROS and impair antioxidant defenses; resultant oxidative stress activates TGF-β signaling and EMT transcriptional programs, fostering epithelial dedifferentiation. Notably, EMT reciprocally suppresses PPAR-mediated lipid pathways, establishing a positive-feedback loop linking lipid disorder, EMT and oxidative stress that accelerates fibrogenesis. Intervention studies show that pharmacological restoration of Scd1 or supplementation with lipid-metabolic intermediates partly reverses EMT phenotypes and fibrosis, highlighting fatty-acid metabolism as a potential target.

Conversely, Shin et al. uncovered a crucial role for CPT1A-driven fatty-acid oxidation (FAO) in sustaining metabolic homeostasis of alveolar epithelium (76). CPT1A, the rate-limiting enzyme of FAO, when deficient, impedes mitochondrial fatty-acid uptake, resulting in ROS build-up, loss of membrane potential and heightened oxidative stress. FAO inhibition markedly potentiates TGF-β1-induced EMT, evidenced by reduced E-cadherin and increased α-SMA and Vimentin. In bleomycin-challenged mice, reduced CPT1A correlates with intensified collagen deposition, higher fibrosis scores and impaired lung function (77). Furthermore, CPT1A loss provokes mitochondrial dysfunction and lipid peroxidation that activate ferroptosis, thereby aggravating alveolar damage and advancing fibrosis. To provide a concise overview, the interconnections between metabolic reprogramming, EMT, and pulmonary fibrosis are summarized in Table 1, highlighting the key pathways, molecular regulators, experimental evidence, and therapeutic implications.

**Table 1:** Summary of metabolic reprogramming pathways driving EMT in pulmonary fibrosis.

**Pathway**	**Key molecules**	**Mechanistic link to EMT**	**Models/interventions**	**Implications**	**Ref**.
Glycolysis	HK2, PFKFB3, LDHA, MCT1	Glycolysis↑ → lactate buildup → TGF-β activation → EMT amplification	*In vitro:* LPS ↓MCT1, triggers EMT. *In vivo:* MCT1 overexpression restores homeostasis	MCT1 and glycolytic enzymes as therapeutic targets	(69, 70)
Glutamine metabolism	GLS1, α-KG	Glutamine catabolism activates TGF-β, epigenetic EMT gene regulation	*In vitro:* TGF-β ↑GLS1, promotes fibrosis. *Drug:* CB-839 inhibits collagen synthesis	GLS1 inhibition shows anti-fibrotic potential	(72, 73)
Fatty acid synthesis	Scd1, Elovl6	Loss of lipid enzymes → ROS ↑, EMT phenotype (↓E-cadherin, ↑Vimentin)	*In vivo:* AT2-specific KO causes spontaneous fibrosis. *Drug:* Lipid supplementation partially reverses EMT	Lipid metabolic restoration may counter fibrosis	(75)
Fatty acid oxidation (FAO)	CPT1A	CPT1A deficiency → FAO ↓ → ROS ↑, ferroptosis → EMT enhancement	*In vitro:* CPT1A loss ↑ EMT markers. *In vivo:* BLM mice with CPT1A↓ show severe fibrosis	Restoring FAO may prevent EMT progression	(76)

### 3.3 Mechanical stress and ECM remodeling

Throughout the initiation and progression of pulmonary fibrosis, mechanical stress—an essential exogenous biophysical cue—is regarded as a pivotal regulator of epithelial-to-mesenchymal transition (EMT) and fibrotic tissue expansion (78). Mechanical loading derives principally from alveolar stretch, airflow-induced shear, tissue compression, and stress transmission caused by extracellular-matrix (ECM) stiffening. Under conditions of alveolar damage, ventilatory imbalance, or matrix remodeling, integrins together with focal-adhesion kinase (FAK) sense mechanical cues and transduce them intracellularly, thereby activating several EMT-related pathways (79).

**Figure 3:**
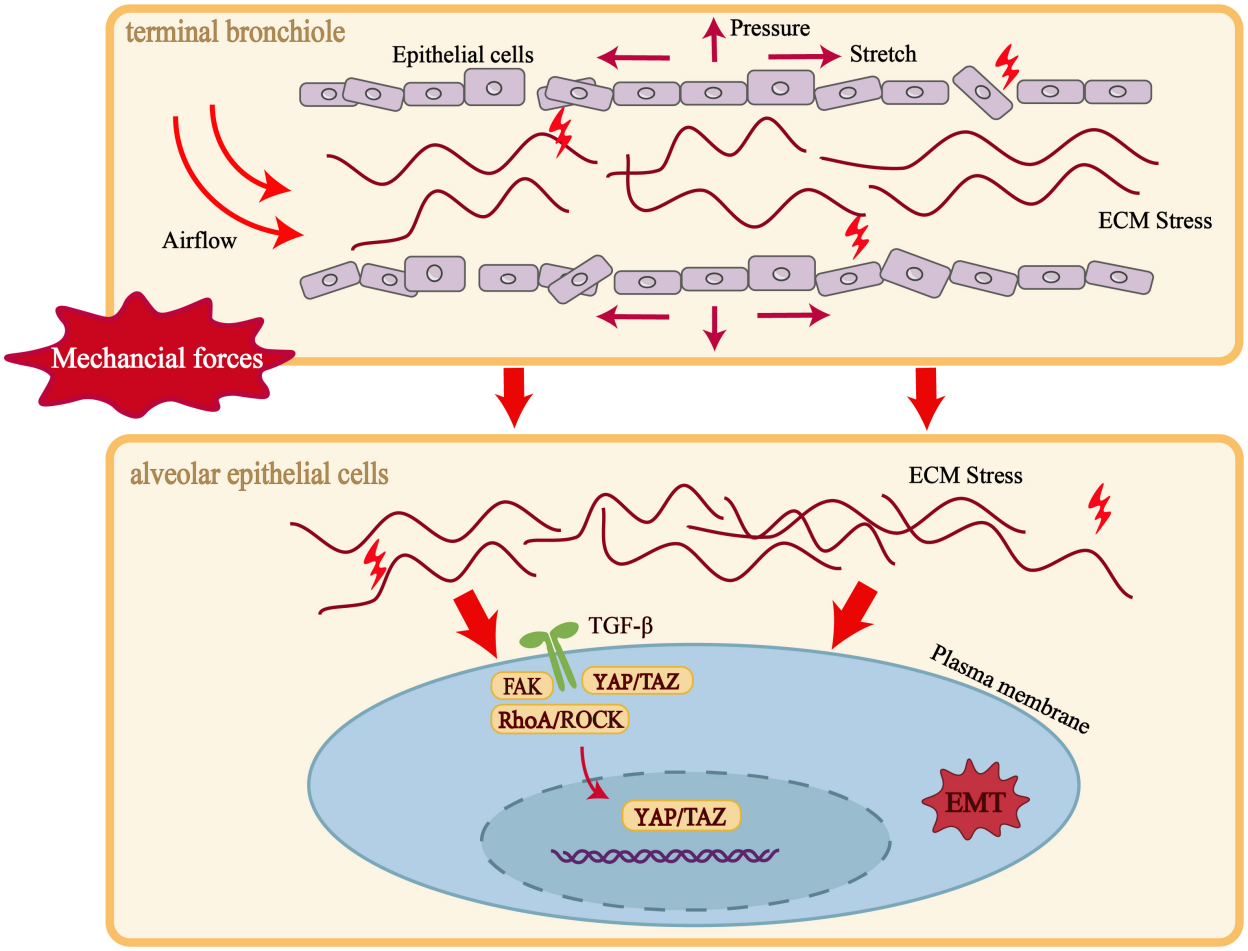
Mechanical stress–ECM remodeling feedback loop driving EMT in pulmonary fibrosis. Mechanical cues derived from alveolar stretch, airflow shear, pressure, and ECM stiffening are sensed by epithelial cells through integrins and transduced via FAK, RhoA/ROCK, and YAP/TAZ signaling. These pathways activate TGF-β signaling, suppress epithelial markers (e.g., E-cadherin), and induce mesenchymal programs (e.g., Vimentin, Snail), thereby promoting EMT and fibrotic remodeling. Newly deposited ECM further increases tissue stiffness, which reinforces mechanotransduction and sustains a self-amplifying “mechanical stress–EMT–fibrosis” feedback loop.

Experimental data demonstrate that sustained mechanical stretch activates the TGF-β/Smad axis, suppresses E-cadherin, and elevates mesenchymal markers such as Vimentin and Snail, thereby driving alveolar epithelial cells toward a mesenchymal phenotype. Cells sense alterations in ECM stiffness and activate mechano-transductive pathways—RhoA/ROCK, FAK/Src, and YAP/TAZ—leading to nuclear translocation of YAP/TAZ, enhanced transcriptional activity, and promotion of the EMT program (80). Concurrently, elevated matrix stiffness—a hallmark of the fibrotic niche—continuously activates mechano-sensitive transcription factors YAP/TAZ, thereby further initiating the EMT transcriptional program. EMT activation compromises epithelial integrity and barrier function while enhancing cellular motility and matrix-synthetic capacity, directly propelling fibrosis. Notably, EMT-induced matrix deposition and tissue stiffening feedback to intensify local mechanical stress, creating a “mechanical stress–EMT–fibrosis” positive-feedback loop that perpetuates pathology. Consequently, targeting mechanotransduction pathways is considered a promising strategy to curb pulmonary fibrosis progression. The overall interplay between mechanical stress, EMT activation, and ECM remodeling is summarized in Figure 3, which highlights the key mechanotransductive pathways and the self-reinforcing feedback loop.

## 4 Pathological links between EMT and pulmonary fibrosis

EMT plays a central regulatory role in pulmonary fibrosis, characterized by remodeling of alveolar-epithelial phenotypes, down-regulation of epithelial markers (e.g., E-cadherin) and up-regulation of mesenchymal markers (e.g., vimentin, N-cadherin), together with fibroblast-to-myofibroblast differentiation that establishes a pro-fibrotic micro-environment and drives disease progression (4). In IPF, AT2 cells—key cells for alveolar regeneration—readily undergo EMT under stimuli such as bleomycin, leading to myofibroblast conversion and collagen deposition (81). Single-cell sequencing has identified an EMT-intermediate epithelial subset in fibrotic lungs that co-expresses COL1A1 and extracellular-matrix genes, implying that alveolar epithelium is not only the starting point of EMT but also a direct contributor to matrix deposition (82).

In pneumoconiosis (e.g., silicosis), AT2 cells similarly undergo EMT but with distinct upstream triggers. In a murine model, Chen et al. demonstrated that silica exposure induces AT2 proliferation and EMT, producing collagen-secreting myofibroblasts; co-exposure to nicotine intensifies this process via STAT3-BDNF-TrkB and Twist signaling (83). This finding suggests that environmental and occupational exposures can activate EMT through specific signaling networks different from those in IPF.

Beyond alveoli, airway epithelial EMT contributes to airway remodeling in both IPF and non-IPF PF subtypes (84). For instance, combined TNF-α + TGF-β1 activates the NF-κB/NOX4 axis in BEAS-2B cells, repressing E-cadherin while increasing α-SMA and vimentin, thereby conferring migratory and ECM-synthetic capacities (85). In cigarette-smoke extract (CSE)-induced *in-vitro* models, airway epithelial cells undergoing CSE-driven EMT and TGF-β1/SMAD3 activation lose epithelial markers (E-cadherin), gain mesenchymal traits (up-regulation of N-cadherin, Slug, α-SMA), exhibit greater motility and ECM synthesis, and markedly elevate fibrotic proteins such as collagen IV and fibronectin-1, thereby further promoting lung fibrosis (86).

During tissue injury in organ fibrosis, EMT's impact becomes increasingly significant, coinciding with decreased epithelial markers and the presence of type I collagen and α-SMA within fibroblasts to enhance matrix deposition. TGF-β directly elevates transcription of collagen genes (COL1A1, COL3A1) via the Smad pathway and induces additional pro-fibrotic mediators (CTGF, PDGF, FGF), synergistically promoting fibroblast proliferation and collagen synthesis (87). EMT-derived myofibroblasts secrete type I/III collagen and fibronectin to form fibroblastic foci (FF)—the key pathological hallmark of IPF; these lesions are activated via Wnt/β-catenin signaling, form through myofibroblast aggregation, involve aberrant epithelial-mesenchymal crosstalk and remodeling, and culminate in fibrosis and honeycombing (88, 89).

EMT not only directly damages alveolar architecture but its heterogeneity is closely associated with clinical differences among disease subtypes. Researchers have shown that alveolar epithelial cells in healthy lungs can undergo dynamic EMT or mesenchymal-to-epithelial transition (MET) (1). In PF lungs, “partial EMT” cells co-expressing E-cadherin and vimentin are present, and EMT-experienced alveolar cells contribute to honeycomb-like fibroblastic foci. Suppressing EMT reduces the formation of new fibrotic foci, whose spatial distribution positively correlates with the degree of tissue remodeling (90).

## 5 Targeting EMT to inhibit pulmonary fibrosis

### 5.1 Small-molecule agents targeting the TGF-β pathway

PF has emerged as a growing global public-health concern, with idiopathic pulmonary fibrosis (IPF) attracting particular attention owing to its high mortality. The TGF-β signaling pathway, a central molecular hub governing EMT, drives fibroblast activation and aberrant extracellular-matrix (ECM) deposition, and has therefore become a major antifibrotic target. Evidence shows that inhibiting the TGF-β pathway with small-molecule inhibitors, neutralizing monoclonal antibodies, or downstream Smad blockade effectively reverses EMT, suppresses fibroblast-to-myofibroblast differentiation, and thereby modulates aberrant lung repair and fibrotic progression. Furthermore, documented therapeutic strategies for inhibiting EMT and intervening in pulmonary fibrosis are summarized in Table 2.

**Table 2:** Recent progress in the development of anti-pulmonary fibrosis therapeutics targeting epithelial–mesenchymal transition (EMT).

**Drug class**	**Intervention**	**Research stage**	**Mechanism**	**Model (animal or human)**	**Refs**.
Small-molecule inhibitor	Pirfenidone (PFD)	IPF (approved); pulmonary fibrosis (investigated)	Inhibits Wnt/GSK-3β/β-catenin Suppresses TGF-β1 and Smad2/3 phosphorylation Blocks the NET–NLRP3 axis	Bleomycin-induced pulmonary fibrosis mouse model	(91, 92)
	Nintedanib	IPF, PF-ILD (approved); pulmonary fibrosis (investigated)	Antagonizes PDGFR/FGFR/VEGFR Inhibits PI3K/Akt/mTOR	Bleomycin-induced pulmonary fibrosis mouse model	(93)
	Nerandomilast	IPF (investigational)	Selective PDE4 inhibition Inhibits PI3K/Akt/NF-κB axis Suppresses TGF-β1/Smad3 and ERK	Multicenter, randomized, placebo-controlled phase III trial in IPF patients	(94, 95)
	Galunisertib	Radiation-induced lung fibrosis (investigated)	Interferes with TGF-β/Smad signaling	Thoracic irradiation (20 Gy)–induced pulmonary fibrosis mouse model	(96)
Natural compound	Triptolide	Pulmonary fibrosis (IPF model; investigated)	Blocks IGF-1-mediated EMT Inhibits TGF-β1/Smad2/3	Bleomycin-induced pulmonary fibrosis mouse model	(99, 102, 103)
	Astragalus saponins (ASTs)	Pulmonary fibrosis (IPF model; investigated)	Inhibits TGF-β1/Smad2/3	Bleomycin-induced pulmonary fibrosis rat model	(104–106)
	Wogonin	Pulmonary fibrosis (IPF model; investigated)	Binds TGF-βRI Inhibits TGF-β/Smad pathway	Bleomycin-induced pulmonary fibrosis rat model	(108)
Gene therapy	miR-125b-5p	Pulmonary fibrosis (IPF model; investigated)	Down-regulates BAK1 Inhibits EMT	Bleomycin-induced pulmonary fibrosis mouse model	(110)
	miR-326	Pulmonary fibrosis (silica-induced model; investigated)	Targets NFIB 3′-UTR, restoring NFIB Inhibits TGF-β signaling	Silica dust–induced pulmonary fibrosis mouse model	(111)
Transcription-axis inhibitor	MSI2-ZEB1 axis inhibition	Radiation-induced lung fibrosis (investigated)	Down-regulates RNA-binding protein MSI2 Directly silences ZEB1	Radiation-injured murine alveolar epithelial cell model	(115)

Pirfenidone (PFD) is a representative antifibrotic drug currently approved for treating IPF. Studies show that PFD not only alleviates pulmonary inflammation and collagen deposition but also exerts antifibrotic effects through multiple key signaling pathways. Mechanistically, PFD inhibits phosphorylation of GSK-3β within the Wnt/GSK-3β/β-catenin axis, reduces β-catenin accumulation, and thereby blocks Wnt-driven fibrogenesis and EMT; it also suppresses activation of TGF-β1 and downstream Smad2/3, diminishing ECM deposition and preventing EMT initiation (91). Recent work shows that, in a myositis-associated interstitial-lung-disease (MAILD) mouse model, PFD markedly suppresses neutrophil-extracellular-trap (NET) formation and blocks NET-mediated activation of the NLRP3 inflammasome, thereby lowering pro-inflammatory cytokines IL-1β, IL-6 and TNF-α and attenuating pulmonary inflammation. Moreover, PFD effectively suppresses NET- and NLRP3-mediated EMT, prevents fibroblast activation and excessive collagen deposition, and ultimately retards pulmonary-fibrosis progression (92).

Nintedanib, another orally administered small-molecule used clinically for IPF, is a multitarget tyrosine-kinase inhibitor that primarily blocks the platelet-derived (PDGFR), fibroblast growth factor (FGFR) and vascular endothelial growth factor (VEGFR) receptor pathways. Beyond its classical anti-angiogenic and anti-proliferative actions on fibroblasts, recent studies reveal that Nintedanib exerts antifibrotic activity by modulating the epithelial–mesenchymal transition (EMT). In bleomycin-induced fibrotic mice and TGF-β1-challenged A549 cells, Nintedanib restores E-cadherin while reducing vimentin and α-SMA, chiefly by suppressing the PI3K/Akt/mTOR axis to halt TGF-β1-driven migration, trans-differentiation and ECM deposition (93).

By contrast, Nerandomilast, a novel selective phosphodiesterase-4 (PDE4) inhibitor, has recently attracted considerable attention in pulmonary-fibrosis research. Studies demonstrate that the drug markedly attenuates alveolar destruction and collagen deposition in bleomycin-treated mice while modulating multiple fibrosis-related pathways. Mechanistically, Nerandomilast alleviates the chronic inflammatory milieu of lung tissue by blocking PI3K/Akt/NF-κB signaling and down-regulating pro-inflammatory mediators (e.g., IL-1β, TNF-α), thereby indirectly curbing inflammation-driven EMT activation (94). In addition, Nerandomilast concurrently suppresses TGF-β1/Smad3 and ERK signaling, markedly reverses TGF-β1-induced EMT, restores the epithelial phenotype and down-regulates mesenchymal markers (95). Collectively, Nerandomilast offers a broader antifibrotic approach by simultaneously targeting canonical and non-canonical EMT pathways.

Galunisertib is a highly selective inhibitor of transforming growth factor-β receptor I (ALK5) that specifically targets the TGF-β/Smad signaling cascade. Studies show that Galunisertib markedly attenuates bleomycin-induced pulmonary fibrosis in mice, an effect that relies predominantly on inhibition of TGF-β/Smad signaling. The compound blocks TGF-β1-induced phosphorylation of Smad2/3, effectively represses the epithelial-to-mesenchymal transition (EMT), prevents alveolar epithelial cells from adopting a pro-fibrotic phenotype, and consequently reduces collagen deposition and fibrotic progression (96).

### 5.2 Natural compounds

In recent years, numerous natural products have shown distinctive therapeutic potential for targeting EMT and modulating the pathological course of pulmonary fibrosis; these bioactive molecules act on multiple targets—including EMT-related transcription factors and key signaling cascades—to effectively block epithelial-mesenchymal conversion, suppress myofibroblast activation and regulate aberrant extracellular-matrix (ECM) remodeling, thereby markedly curbing EMT progression and fibrotic disease (97, 98).

*Triptolide* (TP), an active diterpenoid extracted from Tripterygium wilfordii Hook F, possesses anti-inflammatory, antiproliferative and immunomodulatory properties, and has been widely investigated in experimental idiopathic pulmonary fibrosis (IPF) models in recent years (99, 100). Multiple experiments demonstrate that TP significantly alleviates bleomycin-induced lung fibrosis in mice, restoring alveolar architecture and reducing inflammatory infiltration and collagen deposition (101, 102). Cell-culture data show that TP reverses TGF-β1-induced EMT in A549 cells by restoring E-cadherin and lowering α-SMA, vimentin and fibronectin, thereby inhibiting the transdifferentiation of epithelial cells into a fibroblastic phenotype. Mechanistic analyses reveal that TP suppresses the TGF-β1/Smad2/3 axis, blocking Smad2/3 phosphorylation and nuclear translocation, which in turn halts downstream transcriptional activation, further limits ECM production and myofibroblast accumulation, and attenuates tissue remodeling (103).

Total saponins of *Astragalus membranaceus* (ASTs) are the principal active constituents isolated from *Astragalus membranaceus*; numerous bleomycin (BLM) models have validated their antifibrotic efficacy (104). Evidence indicates that ASTs markedly attenuate inflammation and collagen deposition in BLM-treated mice, thereby ameliorating structural abnormalities (105). The core antifibrotic mechanism lies in suppressing TGF-β1-induced epithelial–mesenchymal transition (EMT), evidenced by restoration of epithelial markers and down-regulation of mesenchymal phenotypes. At the molecular level, ASTs inhibit activation of the TGF-β1/Smad2/3 pathway, thereby blocking EMT signaling and extracellular-matrix deposition, and consequently retarding pulmonary-fibrosis progression (106).

Wogonin, a principal flavonoid constituent of *Scutellaria baicalensis*, effectively mitigates bleomycin-induced pulmonary fibrosis in mice, suppresses inflammatory responses, and restores alveolar architecture. Investigations reveal that Wogonin significantly suppresses TGF-β1-induced epithelial–mesenchymal transition (EMT), reinstating E-cadherin expression while reducing mesenchymal markers. Mechanistically, Wogonin acts chiefly by inhibiting TGF-β1/Smad3 signaling, diminishing Smad3 phosphorylation and nuclear translocation, thereby halting EMT progression and extracellular-matrix deposition (107). Furthermore, UPLC-Q-TOF-MS profiling and molecular docking confirm that Wogonin is a major *S. baicalensis* component capable of stably binding TGF-β receptor I, supporting its direct modulation of this pathway (108).

In summary, several natural compounds have shown the capacity to attenuate pulmonary-fibrosis progression in experimental models by targeting the TGF-β-driven EMT pathway, thereby offering novel therapeutic candidates and mechanistic targets for antifibrotic intervention.

### 5.3 Biologics and gene therapy

In the realm of RNA-based interventions, recent studies indicate that microRNAs (miRNAs) have emerged as novel therapeutic targets; by directing the activation of fibroblasts, EMT, and ECM remodeling, miRNAs exert pivotal regulatory control over fibrotic disease progression, and this multi-pathway capability positions them as a major focus of antifibrotic research (109). Zhou et al. demonstrated that in bleomycin-induced pulmonary-fibrosis models, miR-125b-5p specifically suppresses EMT in lung epithelial cells and attenuates disease severity; over-expression of miR-125b-5p lowers the pro-apoptotic gene BAK1, diminishes EMT proteins (N-cadherin, vimentin) and elevates the epithelial marker E-cadherin, thereby mitigating fibrosis (110). Notably, NFIB expression is markedly reduced in IPF patients and BLM-treated mice; miR-326 targets the 3′-UTR of NFIB, restores its expression, and NFIB up-regulation suppresses TGF-β signaling, lowers collagen deposition and mesenchymal markers, thereby reversing fibrotic pathology (111). siRNA, shRNA and microRNA play crucial roles in regulating EMT by silencing EMT-related genes or augmenting endogenous miRNA expression, thereby constraining the dynamic EMT program. Nanotechnology-based carriers furnish powerful tools for targeted RNA delivery, improving tissue specificity and bioavailability and enabling more precise intervention in EMT (112).

Recently, additional mechanistic targets have been identified: Piezo1, a stretch-sensitive non-selective cation channel, mediates Ca^2^^+^ influx and initiates downstream mechanotransduction. Evidence indicates that Piezo1 amplifies EMT by inducing Ca^2^^+^ influx and co-activating RhoA/ROCK1 and p38-MAPK pathways, culminating in collagen deposition and pulmonary fibrosis (113). Accordingly, targeting Piezo1 and its downstream pathways is regarded as a highly promising EMT-oriented therapy for ventilation-related and other stress-induced pulmonary fibrosis (114). Additionally, ionizing radiation markedly elevates the RNA-binding protein MSI2 in AT2 cells, triggering ZEB1-driven EMT, collagen deposition and radiation-induced pulmonary fibrosis. Cai et al. proposed an “MSI2–ZEB1 axis inhibition” strategy that targets MSI2 or directly suppresses ZEB1, offering a novel anti-EMT intervention to halt fibrosis and potentially synergise with antioxidative or anti-TGF-β therapies, thereby opening translational avenues for radiation-induced and other forms of pulmonary fibrosis (115).

## 6 Challenges and outlook

### 6.1 Limitations of current studies

Although substantial progress has been made in recent years in elucidating the role of epithelial-to-mesenchymal transition (EMT) in the pathogenesis of pulmonary fibrosis, the field is still hampered by numerous controversies and technical bottlenecks that require systematic investigation and methodological innovation. First, there is still no unified standard for detecting EMT. Because EMT is a dynamic and continuous process, current assessments rely largely on immunohistochemistry or Western blot analysis of epithelial and mesenchymal markers such as E-cadherin, α-SMA and vimentin. However, some cells may display a hybrid phenotype during transition, making it difficult to determine unequivocally whether EMT has occurred. Therefore, lineage tracing and single-cell omics technologies (e.g., scRNA-seq) provide more precise means to determine whether specific cell populations undergo genuine phenotypic conversion.

Second, limitations inherent to animal models constrain the translational relevance of current findings. Although the bleomycin-induced mouse model is widely employed to study EMT and pulmonary-fibrosis mechanisms, it chiefly mirrors acute injury–repair cycles and differs markedly from the chronic, progressive nature of human IPF. In clinical practice, pulmonary fibrosis represents a heterogeneous spectrum of phenotypes and subtypes, characterized by diverse etiological factors and pathological mechanisms. Idiopathic pulmonary fibrosis (IPF), characterized by the UIP pattern, is considered the prototypical form and is frequently modeled in mice through bleomycin administration or TGF-β overexpression (116). The progressive fibrosing phenotype (PPF) occurs across multiple ILDs and is typically reproduced in animal studies using chronic or repetitive low-dose bleomycin exposure (117, 118). In CTD-associated ILDs, systemic sclerosis is commonly studied using Fra-2 transgenic mice or HOCl-induced fibrosis (119), while RA-associated ILD is generally replicated through SKG mice with arthritis induction (120). Among occupational pneumoconioses, silicosis is generated by crystalline silica exposure (121), and asbestosis is induced through inhalation or instillation of asbestos fibers (122). While these models cannot entirely reproduce the chronic and heterogeneous nature of human disease, they remain indispensable, offering complementary platforms for mechanistic insights and preclinical antifibrotic drug development.

Third, the concept that EMT can be reversed is still debated, given the paucity of direct *in vivo* evidence (123), with some studies suggesting the occurrence of mesenchymal-to-epithelial transition (MET), whereas others argue that fibrosis resolution is mainly driven by myofibroblast apoptosis rather than phenotypic reversion (124). Recent lineage-tracing studies suggest that myofibroblast depletion during fibrosis resolution results mainly from apoptosis rather than phenotypic reversion, challenging antifibrotic strategies predicated on “reversible EMT”. Finally, organ- and region-specific differences further complicate mechanistic dissection. EMT-regulatory mechanisms may differ substantially among organs (lung, kidney, liver), and even within the lung, alveolar and bronchial epithelia display divergent responses and EMT potentials to TGF-β. Accordingly, elucidating lung-specific EMT circuitry and microenvironment-dependent modulation, and validating findings across PF subtypes, will be a crucial direction for future research.

### 6.2 Future research directions

To facilitate the clinical translation of EMT-based mechanistic insights in pulmonary fibrosis, systematic investigations can be pursued along several directions. Integrating spatiotemporal omics technologies—such as single-cell transcriptomics, epigenomics and spatial transcriptomics—will enable comprehensive mapping of the evolutionary trajectories of EMT-related cells and their local micro-environmental interaction networks during fibrogenesis, thereby identifying regulatory nodes and therapeutic targets. Patient-derived organoids and lung-on-a-chip devices that recapitulate three-dimensional architecture and mechanical stress provide high-fidelity platforms for dissecting EMT mechanisms and for drug screening. Integrating multi-omics datasets—including genomics, transcriptomics, proteomics and metabolomics—will aid in constructing molecular network maps of EMT regulation, reveal synergistic crosstalk among signaling pathways, and support the development of combination-therapy strategies. Developing lung-targeted drug-delivery systems—such as nanoparticles and liposomes—could enhance pulmonary accumulation and bioavailability of anti-EMT agents, while reducing systemic toxicity and improving therapeutic precision and safety. For EMT regulation involving multiple pathways and cell types, exploring polypharmacology or multi-drug regimens may improve intervention efficacy.

It is noteworthy that metabolic dysregulation bears a potential link to pulmonary fibrosis. A meta-analysis (26 million individuals) reported a 54% increased risk of diabetes in patients with pulmonary fibrosis, suggesting that insulin resistance, lipotoxicity and other metabolic abnormalities may promote fibrosis via EMT. Elucidating the interplay between metabolic signaling and EMT may yield more targeted therapeutic strategies for pulmonary-fibrosis patients with comorbid metabolic disorders.

## 7 Conclusions

EMT is a pivotal process in pulmonary fibrosis (PF). It is regulated by the TGF-β superfamily, inflammatory mediators, Wnt/β-catenin and Hippo–YAP/TAZ pathways. New mechanisms have expanded the understanding of PF pathology. Several preclinical agents reduce fibrosis and EMT in cell and animal models. However, these agents have not yet been tested in clinical trials.

Uncertainties remain about the exact role of EMT in PF. Its contribution may vary between full and partial EMT, and between IPF and non-IPF fibrotic ILDs. Paracrine signaling between epithelial cells and fibroblasts also needs clarification. Current detection methods often rely on a few markers, such as E-cadherin, α-SMA and vimentin, which do not fully capture transitional cell states or spatial heterogeneity.

Future studies should focus on several priorities. First, develop standardized EMT assessment methods using multiple markers, lineage tracing and single-cell or spatial omics. Second, verify mechanisms in human-relevant models, such as explanted lung tissue, precision-cut lung slices and patient-derived organoids. Third, identify and validate biomarkers to classify PF subtypes and monitor treatment response. Fourth, design translation-ready studies that combine lung-targeted drug delivery, target engagement assessment and early-phase trials stratified by disease subtype. Linking mechanistic EMT modulation with patient outcomes may enable precise, stage-specific intervention. This could open new therapeutic strategies for pulmonary fibrosis.
